# Utilizing SMOTE-TomekLink and machine learning to construct a predictive model for elderly medical and daily care services demand

**DOI:** 10.1038/s41598-025-92722-1

**Published:** 2025-03-11

**Authors:** Guangmei Yang, Guangdong Wang, Leping Wan, Xinle Wang, Yan He

**Affiliations:** 1 The Affiliated Encephalopathy Hospital of Zhengzhou University, Zhumadian, Henan China; 2 Zhengzhou University, Zhengzhou, Henan China; 3 Northwest Agriculture and Forestry University College of Natural Resources and Environment, Xianyang, Shaanxi China; 4 Wuhan University, Wuhan, Hubei Province China; 5 Hainan Medical University, Haikou, Hainan China

**Keywords:** SMOTE-TomekLink, Machine learning, The elderly, Predictive model, Medical and daily care services demand, Risk factors, Geriatrics, Public health

## Abstract

This study aims to construct a prediction model for the demand for medical and daily care services of the elderly and to explore the factors that affect the demand for medical and daily care services of the elderly. In this study, a questionnaire survey on the demand for medical and daily care services of 1291 elderly was conducted using multi-stage stratified whole cluster random sampling. SPSS21.0 statistical analysis software was used to describe the basic data of the elderly statistically, and univariate analysis was used to screen variables for model construction and binary logistic regression analysis. The acquired dataset has class imbalance, and to handle this issue, Synthetic Minority Over Sampling Technique with TomekLink (SMOTE-TomekLink) was adopted to resample the dataset for class-balancing. To improve computational efficiency, we used three algorithms to develop prediction models, including Random Forest (RF), Gradient Boosting Decision Tree (GBDT), and Light Gradient Boosting Machine (LightGBM) algorithms. The performance of each model was measured, and the performance of the prediction model was obtained using the following performance metrics: accuracy (ACC), recall (R), precision (P), F1-score, and area under the receiver operating characteristic (AUC). The prediction models for the medical and daily care services demand of the elderly were developed and validated using 12 and 13 key features, respectively. The LightGBM algorithm emerged as the superior prediction model for estimating the service needs of the elderly. For the medical service demand prediction model, LightGBM achieved an AUC of 0.910 and F1-score of 0.841. In the daily care services demand prediction model, LightGBM demonstrated an AUC of 0.906 and an F1-score of 0.819. In the LightGBM model, the analysis of feature importance indicates that the number of chronic diseases, education level, and financial sources emerge as the most significant predictors for the demand of healthcare services, encompassing both medical and daily care services. Based on questionnaire information combined with feature selection, unbalanced data processing and machine learning methods, this study constructed a machine learning model for predicting the demand for medical and daily care services for the elderly, and analyzed the influencing factors of the demand for medical and daily care services for the elderly, providing a reference for the construction and verification of future prediction models for the demand for medical and daily care services for the elderly.

**Supplementary Information:**

The online version contains supplementary material available at 10.1038/s41598-025-92722-1.

## Introduction

The phenomenon of population aging is a hot topic of concern worldwide. According to the criteria for the division of aging set out in the United Nations’ “Population Ageing and its Socio-Economic Consequences,” China entered an aging society at the beginning of the 20th century. Over the following two decades, China’s aging population increased significantly, and the degree of aging of the population became increasingly prominent. According to China’s seventh census, there are 264.02 million elderly people aged 60 and above, accounting for 18.7% of the total population^1^. As the population ages, the number of elderly with disabilities, semi-disabilities, and chronic diseases will increase.

These vulnerable populations require not only medical services but also daily care services. Medical services for the elderly refer to those tailored to meet the specific health needs of older adults. This includes, but is not limited to, routine medical check-ups, disease prevention, diagnostic services, treatment of acute and chronic illnesses, rehabilitation services, and end-of-life care. These services are crucial in managing the multiple health challenges that often accompany aging. Daily care services encompass a broader range of support services aimed at maintaining or improving the quality of life for elderly individuals. This includes assistance with activities of daily living (ADLs), such as bathing, dressing, eating, and mobility, as well as social and emotional support to combat loneliness and isolation. Daily care services can be provided in various settings, including the home, community centers, or specialized care facilities. The long-term care system in the United States offers insights into addressing the challenges of eldercare, yet it faces a persistent shortage of care workers due to the growing elderly population. According to the Health Resources and Services Administration, the demand for direct care workers for the elderly is projected to increase by 34%, necessitating an additional 650,000 workers^2^. Similarly, studies in other developing countries have shown that as the aging population rises, the growing demand for home care services also increases^3^. In China, the prevalent “4-2-1” family structure in China, where two working adults are responsible for the care of four elderly parents and one child, is straining the traditional model of family-based eldercare. This family structure is becoming increasingly unsustainable^4^. Furthermore, the high costs and insufficient supply of institutional eldercare, coupled with the elderly’s strong preference for family care, prevent institutional care from becoming the dominant model^5,6^. China’s demographic landscape is unique in that it is aging before it becomes wealthy, which adds significant pressure to the country’s eldercare security system. In light of these challenges, it is imperative for China to focus on the escalating demand for medical and daily care services among the elderly. This attention is crucial for developing comprehensive and sustainable strategies to support the aging population.

Currently, domestic and foreign studies related to the demand for medical services or daily care services for the elderly are primarily focused on analyzing the current situation and the influencing factors^7–9^. The studies still use traditional logistics regression, and there are fewer studies related to the construction of predictive models using machine learning. Machine learning represents a primary approach to data mining. By constructing machine learning models, complex relationships can be automatically processed, and machine models have demonstrated predictive utility in the medical field^10–12^. Additionally, machine learning models have been extensively utilized in other research domains^13–15^. Currently, there is a lack of studies on the demand for healthcare services despite the prevalence of model prediction studies. Previous studies have demonstrated that the performance of a single weak learner can be enhanced by integrating multiple learners. With the application of large-scale data and the advancement of computers technology, machine learning plays a more pivotal role in health risk assessment. A comparison of several studies revealed that RF (Random Forest), GBDT (Gradient Boosting Decision Tree), and LightGBM (Light Gradient Boosting Machine) exhibited superior prediction performance and broader applicability^16–18^. Therefore, this study selected the three models for the construction of a prediction model for the demand for medical and daily care services for the elderly and evaluation and comparison of the prediction performance of the three models. This was done to identify a more suitable model for predicting the demand for medical and daily care services for the elderly.

## Materials and methods

### Study design, setting, and participants

In this study, questionnaire surveys were conducted among community-dwelling elderly individuals aged 60 years or older in three cities: Guangzhou, Suzhou, and Qingdao. These cities were selected using a multi-stage stratified whole cluster random sampling method to ensure the representativeness of the sample. First, one county/district was randomly selected in each city jurisdiction. Yuexiu District was selected from Guangzhou City, Kunshan District from Suzhou City, and Jimo District from Qingdao City. Secondly, two streets were randomly selected from each county/district. Finally, four communities were randomly selected from each street. 1,380 questionnaires were distributed, and 1,291 were returned, with an effective recovery rate of 93.6%. This study was ethically reviewed by Zhengzhou University (ZZUIRB2022-07). All participants provided written informed consent. All methods were carried out following the principles of the Declaration of Helsinki.

### Measures and variables

The questionnaire survey method is a method to measure the problem under study and collecting information using controlled measurements. This study used a self-designed questionnaire to collect demographic characteristics, family resources, socioeconomic status, lifestyle habits, number of chronic diseases, and demand for healthcare (medical and daily care) services for the elderly living at home in communities.

1. Demographic characteristics include age and gender.
2. Socioeconomic status encompasses educational level, financial sources, monthly income, and medical insurance.
3. Family resources included the following variables: spouse, living style, number of children, and frequency of visits.
4. Lifestyle habits include smoking, drinking, and sleep time.
5. Chronic Disease Conditions: This study investigated the types of chronic disease conditions suffered by the elderly, including cerebrovascular diseases, cardiovascular diseases, respiratory diseases, endocrine system and nutritional, metabolic diseases, musculoskeletal diseases, urological diseases, rheumatologic diseases, oncologic diseases, and other diseases. The total number of chronic diseases in older adults was obtained by summing the number of all self-reported diseases in older adults.

In this study, the number of chronic diseases among the elderly was categorized as 0, 1, 2, and ≥ 3 chronic diseases.

The dependent variable in this study was the demand for healthcare services, which encompassed both medical services and daily care services. In this research, medical services referred to individuals willing to receive any of the following: clinical treatment, medical rehabilitation, nursing care, emergency medical assistance, home-based medical care, traditional Chinese medicine services, and preventive health care. Daily care services, referred to individuals open to accepting any of these services: home visits, meal assistance, bathroom assistance, bathing assistance, mobility assistance, emergency response assistance, psychological care, and recreational activities.

In this survey, the utilization of medical and daily care services by the elderly was quantified and categorized based on the number of services required. The demand for medical services among the elderly was categorized based on the number of distinct services required: ‘0’ indicating no demand for medical services, ‘1’ indicating a demand for one type of medical service, ‘2’ indicating a demand for two types of medical services, and ‘≥3’ indicating a demand for three or more types of medical services. Similarly, the demand for daily care services was classified as follows: ‘0’ for no demand, ‘1’ for a demand for one type of daily care service, ‘2’ for a demand for two types of daily care services, and ‘≥3’ for a demand for three or more types of daily care services. For analytical purposes, the frequency of usage of medical services among the elderly participants was categorized into two groups: ‘0’ indicating no demand for medical services, and ‘1’ indicating any demand for medical services. Similarly, the frequency of usage for daily care services was also categorized into two groups: ‘0’ representing no demand for daily care services, and ‘1’indicating any demand for daily care services.

### Feature selection and data preprocessing

Data preprocessing involves organizing data before analyzing them and using them to train models^19^. We preprocessed the dataset to prepare 14 candidate features based on previous studies. Features with a missing percentage of not more than 30% were retained and filled in with mean interpolation. Dataset outliers were addressed by excluding extreme values in the top and bottom 1%. Because the range of different feature values widely varied and some of the used algorithms require standardization of the data to ensure that the variables contribute equitably to the model in subsequent analyses, univariate analysis was used to screen for overall features.

### Machine learning algorithms

1. Random Forest (RF)The RF algorithm was derived from Breiman L^20^, is a widely used ensemble learning method known for its ease of implementation in both classification and regression tasks. It operates by constructing a collection of decision trees, with the final output being determined by a vote among them (for classification) or an average (for regression). In this study, the following parameters were chosen (partially) for the RF model:
  ① Number of trees (ntree = 200): We selected 200 trees to balance computational efficiency with model performance, as this number provided adequate accuracy without excessive computation.
  ② Minimum number of decision tree nodes (nodesize = 1): This parameter is set to 1, allowing the trees to grow until each leaf node contains at least one instance.
2. GBDT (Gradient Boosting Decision Tree) GBDT is an iterative algorithm that builds upon the decision tree framework. It sequentially combines weak learners to form a strong predictive model. The parameters for the GBDT model in this study are as follows (partially):
  ① Learning rate (shrinkage = 0.01): The shrinkage parameter, or learning rate, is set to 0.01 to ensure that each new tree corrects the errors of the previous ones in small increments, which can lead to better convergence and generalization.
  ② Depth of decision tree (interaction.depth = 3): The interaction depth of the trees is limited to 3 to control the model’s complexity and prevent overfitting, while still allowing the model to capture relevant interactions among features.
  ③ Number of iterative regression trees (n.trees = 1 1000): The model uses 1000 trees to iteratively refine its predictions. This number is chosen to provide sufficient model capacity while remaining computationally feasible.
3. LightGBM (Light Gradient Boosting Machine) The LightGBM algorithm was proposed by Ke et al. in 2017^21^, is designed to address the computational inefficiencies of traditional GBDT algorithms. Compared with models such as GBM^22^ and XGBoost^23^, LightGBM effectively addresses the issue of slow computation speed and large memory consumption. The parameters for the LightGBM model in this study are (partially):
  ① Learning rate (1eaming_rate-0 0.125): The learning rate is set to 0.125, which is relatively high and allows for faster convergence during training by making larger updates to the model’s predictions.
  ② Maximum depth of tree model (max_depth = 5): The maximum depth is set to 5 to prevent the model from becoming too complex, which could lead to overfitting, while still allowing it to capture complex patterns in the data.
  ③ Number of iterations for boosting (n_estimators = 1000): Similar to GBDT, 1000 iterations are used to ensure the model has enough capacity to learn from the data without excessive computational cost.
  ④ Number of leaf nodes in a tree (num_1eaves 31): The default value for num_leaves is set to 31, which determines the complexity of the tree. This value is chosen to balance the model’s ability to fit the training data with the risk of overfitting.

### Model development

**Fig. 1:**
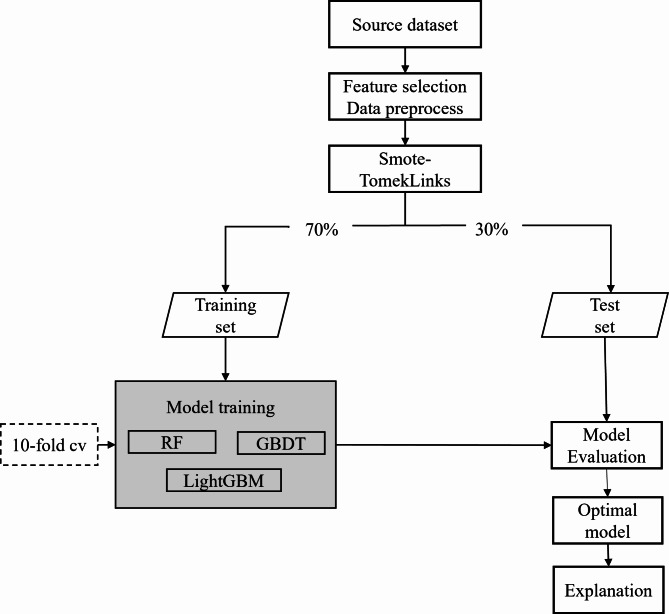
Model development process.

We developed three machine learning models using the filtered features as independent variables and whether an individual has a demand for medical services and a demand for daily care services as dependent variables. Synthetic Minority Over Sampling Technique with TomekLink (SMOTE-TomekLink) was adopted to resample the dataset for class-balancing. The processed dataset was divided into training and test sets in a ratio of 7:3, where the training set was used for model training and the test set was used for model validation. We employed random search to explore different hyperparameter combinations and identify the optimal configuration. A 10-fold cross validation was performed using the training set where one tenth of the training set was used for testing and each part of the remaining nine tenths was used sequentially for training in the training phase for better performance. Confusion matrix is used to evaluate the performance of the model based on ML algorithm. The performance of the model was assessed by calculating the accuracy (ACC), precision (P), recall (R), F1-score, and receiver operating characteristic (ROC) curve and AUC. The development process for the classification prediction models is shown in Fig. 1.

#### SMOTE and TomekLinks for balancing the dataset

The rarity of elderly individuals exhibiting no demand for healthcare services constitutes a low-incidence event, leading to a significant imbalance in the dataset. Imbalanced datasets are a common challenge in machine learning, where the number of samples in one class greatly outnumbers the samples in the other class. In our case, the dataset has a limited number of samples representing elderly people without healthcare needs, which can lead to a biased model that predominantly predicts the majority class.

This imbalance poses a significant challenge during the training and evaluation of classification models, as the model may become biased towards the predominant class, leading to suboptimal performance, especially in terms of the minority class. To address this issue, we employed two resampling techniques: the synthetic minority over-sampling technique (SMOTE)^24^ and TomekLinks^25^. SMOTE is an over-sampling strategy designed to increase the number of minority class samples by synthesizing new examples rather than by over-sampling with replacement. TomekLinks, on the other hand, is an under-sampling approach that aims to reduce the number of samples in the majority class by removing instances that are nearest neighbors to samples of the minority class but are misclassified by a simple classifier. The combination of SMOTE and TomekLinks in a sequential manner allows for a more balanced dataset. First, SMOTE is applied to over-sample the minority class, followed by TomekLinks to under-sample the majority class, ensuring that the synthetic samples generated by SMOTE are not immediately removed by the TomekLinks procedure. This two-step approach maintains a diverse set of examples while cleaning the decision boundary, which can lead to a more robust and fair classification model.

#### Model assessment

The predictive model’s performance was evaluated by calculating a series of performance metrics, including accuracy (ACC), recall (R), precision (P), F1-score, and area under the receiver operating characteristic curve (AUC). The binary confusion matrix had been employed to compute these matrices, Table 1.

**Table 1:** Confusion matrix for binary classification models.

True category	Forecast category	
	Negative	Positive
Negative	True Negative (TN)	False Positive (FP)
Positive	False Negative (FN)	True Positive (TP)

Accuracy (ACC) represents the percentage of samples correctly identified by the model out of the total sample size. Recall (R) indicates the percentage of elderly individuals with actual needs correctly identified by the model out of all elderly individuals with actual needs. Precision (P) reflects the percentage of elderly individuals with actual needs among all individuals identified as having needs by the model. The F1-score represents the harmonic mean of precision and recall, balancing the trade-off between the two^26^. The Area Under the Curve (AUC) of the Receiver Operating Characteristic (ROC) curve is a measure of the model’s predictive ability. The AUC ranges from [0 ~ 1], with higher values indicating better predictive performance^27^. An AUC of 0.5 suggests the model has no predictive ability, values between 0.5 and 0.7 indicate low predictive ability, values between 0.7 and 0.9 suggest moderate predictive ability, and values above 0.9 indicate high predictive ability. The calculation formula is as follows:

*ACC* $$\:=\frac{\text{TP+TN}}{\text{TP+FP+TN+FN}}$$

*R*=$$\:\frac{\text{TP}}{\text{TP+FN}}$$

*P*=$$\:\frac{\text{TP}}{\text{FP+TP}}$$

*F1* = 2$$\:\times\:\frac{\text{R}\times\text{P}}{\text{R+P}}$$

Precision and recall are critical indicators of the model’s predictive performance. A low precision rate may result in a high number of false reports, while a low recall rate indicates that a significant number of elderly individuals with actual needs are not detected^28^. Since accuracy, recall, and precision are individual evaluation metrics and may not comprehensively represent the model’s predictive capabilities, the F1-score and AUC values are considered when assessing the predictive performance of the models. In cases where the accuracy, recall, and precision of the three models are comparable, the F1-score and AUC values are taken into account for a more nuanced evaluation. Performance evaluation matrices are computed under the four confusion matrices.

### Statistical analysis

This study used SPSS 21.0 statistical analysis software to clean and analyze the data. Counts were expressed as frequencies (percentages), and comparisons between groups were made using the *χ*^2^ test. Multicategorical variables were encoded as dummy variables, with significant predictors from univariate tests advancing to feature selection for model building and subsequent binary logistic regression analysis. The significance level was set at α = 0.05. SMOTE-TomekLink resampling, classifier creation, and optimization all took place in R studio 4.1.1 (R Development Core Team). The randomforest, gbm, and lightgbm packages in R studio 4.1.1 (R Development Core Team) were employed to analyze the RF, GBDT, and LightGBM, respectively. Model performance was assessed using AUC, F1-score, Accuracy, Precision, and Recall.

## Results

### Participant characteristics

A total of 1,291 elderly individuals were included in this study. Of these, 724 (56.1%) were females and 567 (43.9%) were males. The percentage of elderly individuals requiring medical services is 87.5%, while the percentage requiring daily care services is 70.8%, as shown in Supplementary Material (eTable 1). The univariate analysis of demand for medical care services and demand for daily care services among the elderly showed that factors such as age, educational level, financial sources, monthly income, medical insurance, spouse, living style, number of children, frequency of visits, smoking, sleep time and number of chronic diseases were statistically significant in differentiating between the groups with and without demand for services (*P* < 0.05). Gender was only statistically significant in differentiating the demand for daily care services among the elderly (*P* < 0.05). As shown in Table 2.

**Table 2:** Demand analysis of medical and daily care services for the elderly.

variables	Total *n*(%)	Demand for medical services		χ^2^	*P*	Demand for daily care services		χ^2^	*P*
		Yes *n*(%)	No *n*(%)			Yes *n*(%)	No *n*(%)		
Gender				3.305	0.069			10.744	0.001
Male	567 (43.9)	507(89.4)	60(10.6)			428(75.5)	139(24.5)		
Female	724 (56.1)	623(86.0)	101(14.0)			486(67.1)	238(32.9)		
Age (years)								182.126	< 0.001
60~	543 (42.1)	448(82.5)	95(17.5)	21.711	< 0.001	287(52.9)	256(47.1)		
70~	327 (25.3)	299(91.4)	28(8.6)			237(72.5)	90(27.5)		
80 ~ 97	421 (32.6)	383(91.0)	38(9.0)			390(92.6)	31(7.4)		
Educational level				15.037	0.002			22.173	< 0.001
Illiterate/Little Literacy	365 (28.3)	312(85.5)	53(14.5)			285(78.1)	80(21.9)		
Primary School	376 (29.1)	350(93.1)	26(6.9)			270(71.8)	106(28.2)		
Middle School	290 (22.5)	247(85.2)	43(14.8)			178(61.4)	112(38.6)		
High school and above	260 (20.1)	221(85.0)	39(15.0)			181(69.6)	79(30.4)		
Financial Sources				14.621	0.002			58.555	< 0.001
Pension	642 (49.7)	567(88.3)	75(11.7)			474(73.8)	168(26.2)		
Relative child allowance	317 (24.6)	290(91.5)	27(8.5)			244(77.0)	73(23.0)		
Labor income	145 (11.2)	116(80.0)	29(20.0)			64(44.1)	81(55.9)		
Other	187 (14.5)	157(84.0)	30(16.0)			132(70.6)	55(29.4)		
Monthly income (Ұ)				10.382	0.016			32.541	< 0.001
≤ 1000	470 (36.4)	401(85.3)	69(14.7)			342(72.8)	128(27.2)		
1001 ~ 3000	353 (27.3)	301(85.3)	52(14.7)			211(59.8)	142(40.2)		
3001 ~ 5000	299 (23.2)	274(91.6)	25(8.4)			237(79.3)	62(20.7)		
≥ 5001	169 (13.1)	154(91.1)	15(8.9)			127(73.4)	45(26.6)		
Medical Insurance				12.159	0.007			37.842	< 0.001
Basic medical insurance for urban and rural workers	371 (28.7)	311(83.8)	60(16.2)			269(72.5)	102(27.5)		
Basic Medical Insurance for Urban and Rural Residents	749 (58.0)	661(88.3)	88(11.7)			519(69.3)	230(30.7)		
Others	104 (8.1)	100(96.2)	4(3.8)			94(90.4)	10(9.6)		
No	67 (5.2)	58(86.6)	9(13.4)			32(47.8)	35(52.2)		
Spouse				21.217	< 0.001			117.506	< 0.001
Yes	878 (68.0)	743(84.6)	135(15.4)			539(61.4)	339(38.6)		
No	413 (32.0)	387(93.7)	26(6.3)			375(90.8)	38(9.2)		
Living Style				28.355	< 0.001			127.698	< 0.001
Lives alone	261 (20.2)	236(90.4)	25(9.6)			224(85.8)	37(14.2)		
Living with family	842 (65.2)	710(84.3)	132(15.7)			510(60.6)	332(39.4)		
Other	188 (14.6)	184(97.9)	4(2.1)			180(95.7)	8(4.3)		
Number of children				16.560	0.001			41.115	< 0.001
0	105 (8.1)	103(98.1)	2(1.9)			94(89.5)	11(10.5)		
1	341 (26.4)	284(83.3)	57(16.7)			212(62.2)	129(37.8)		
2	499 (38.7)	440(88.2)	59(11.8)			338(67.7)	161(32.3)		
≥3	346 (26.8)	303(87.6)	43(12.4)			270(78.0)	76(22.0)		
Frequency of visits				44.320	< 0.001			58.665	< 0.001
Once a day	509 (39.4)	410(80.6)	99(19.4)			304(59.7)	205(40.3)		
Once a week	338 (26.2)	300(88.8)	38(11.2)			259(76.6)	79(23.4)		
Half a month or once a month	296 (22.9)	283(95.6)	13(4.4)			247(83.4)	49(16.6)		
More than once every three months	148 (11.5)	137(92.6)	11(7.4)			104(70.3)	44(29.7)		
Smoking				8.006	0.005			8.753	0.003
Yes	362 (28.0)	798(85.9)	131(14.1)			636(68.5)	293(31.5)		
No	929 (72.0)	332(91.7)	30(8.3)			278(76.8)	84(23.2)		
Drinking				1.074	0.300			0.505	0.477
Yes	315 (24.4)	849(87.0)	127(13.0)			686(70.3)	290(29.7)		
No	976 (75.6)	281(89.2)	34(10.8)			228(72.4)	87(27.6)		
Sleeping time (h)				6.176	0.046			12.245	0.002
< 6	410 (31.8)	368(89.8)	42(10.2)			285(69.5)	125(30.5)		
6 ~ 8	588 (45.5)	500(85.0)	88(15.0)			398(67.7)	190(32.3)		
> 8	293 (22.7)	262(89.4)	31(10.6)			231(78.8)	62(21.2)		
Number of chronic diseases				34.411	< 0.001			135.537	< 0.001
0	225 (17.4)	176(78.2)	49(21.8)			100(44.4)	125(55.6)		
1	355 (27.5)	303(85.4)	52(14.6)			232(65.4)	123(34.6)		
2	306 (23.7)	271(88.6)	35(11.4)			229(74.8)	77(25.2)		
≥3	40531.4	380(93.8)	25(6.2)			353(87.2)	52(12.8)		

### A comparison of the predictive performance of various models

**Fig. 2:**
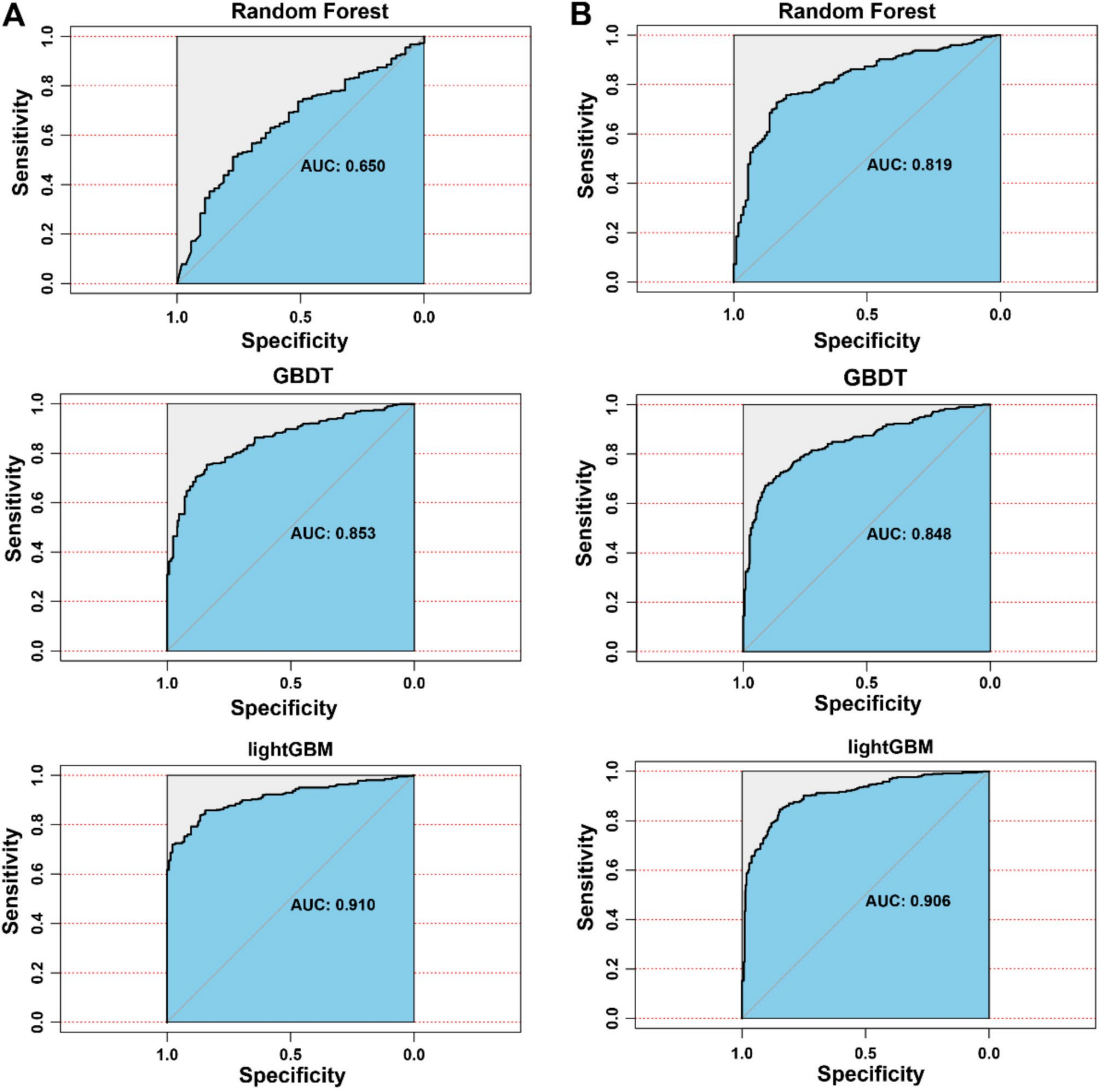
ROC curves for demand forecasting models for healthcare services. Note: A. Comparison of AUC results of medical service demand prediction models. B. Comparison of AUC results of daily care service demand prediction models.

Table 3 presents the prediction performance of three machine learning models across different service demands. The LightGBM model achieves the highest performance for the medical services demand prediction with AUC of 0.910, F1-score of 0.841, ACC of 0.846, R of 0.870 and P of 0.814. On the other hand, the LightGBM model also achieved the best results for the daily care services demand prediction, recording AUC of 0.906, F1-score of 0.819, ACC of 0.830, R of 0.867 and P of 0.777. Figure 2 presents a comparative visualization of the AUC outcomes for the forecasting models pertaining to the demand for medical services and the demand for daily care services.

**Table 3:** Performance comparison of three machine learning models.

Category	Model	ACC	*R*	*P*	F1-score	AUC
Demand for medical services	RF	0.843	0.862	0.973	0.914	0.650
	GBDT	0.787	0.928	0.626	0.747	0.853
	LightGBM	0.846	0.870	0.814	0.841	0.910
Demand for daily care services	RF	0.763	0.803	0.884	0.841	0.819
	GBDT	0.794	0.867	0.648	0.741	0.848
	LightGBM	0.830	0.867	0.777	0.819	0.906

### Importance analysis of the characteristics of each model

**Fig. 3:**
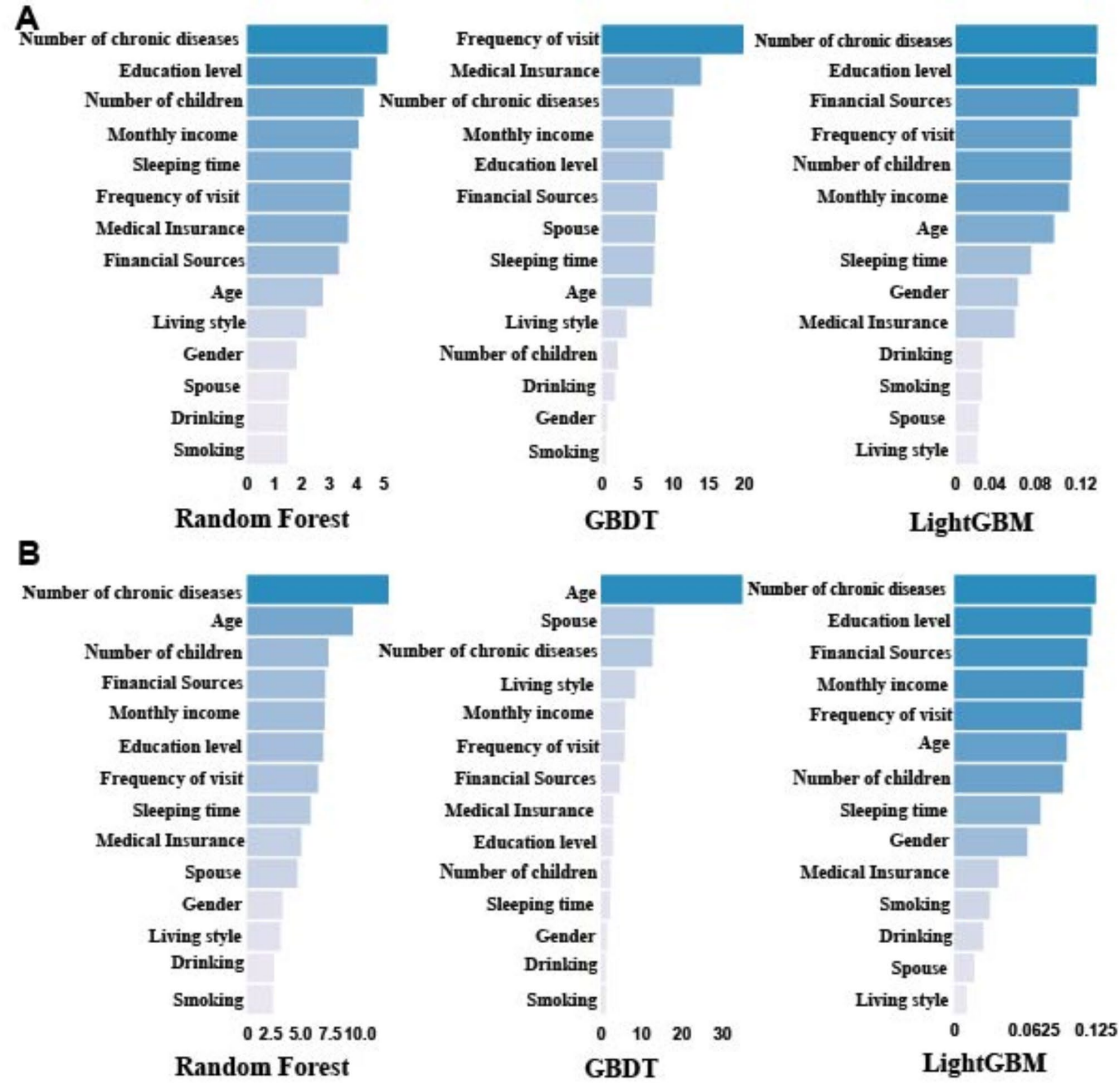
Ranking of the importance of each predictive model feature. Note: A. Ranking of importance of each prediction model in medical service demand prediction. B. Ranking of importance of each prediction model in daily care service demand prediction.

The results of the medical service demand prediction indicated that the top six in the importance ranking of each prediction model were education level, the number of chronic diseases, monthly income and the frequency of visits. The results of the LightGBM model, which had the best prediction performance, demonstrated that number of chronic diseases, education level, and financial source were the most influential factors in determining the demand for medical services among the elderly. As shown in Fig. 3A. The results of the daily care services demand prediction indicated that the top six in the importance ranking of each prediction model were age, the number of chronic diseases, and monthly income. The LightGBM model results demonstrated that the number of chronic diseases, education level, monthly income, and the frequency of visits, had the highest importance in the demand for daily care services for the elderly. As illustrated in Fig. 3B.

## Discussion

Globally, approximately 10.4% of the healthcare and long-term care needs of individuals aged 65 and older remain unmet, with China facing particularly severe challenges^29^. As China’s population ages, the increase in the average age has led to a sharp rise in the demand for healthcare services among the elderly. This study found that among a sample of 1291 elderly individuals selected from three cities in China, the demand for medical services reached 87.5%, and the demand for daily care services reached 70.8%, a proportion that is not commonly observed in previous studies^30,31^, highlighting the importance and urgency of current healthcare service needs. In light of this, the present study sampled 1291 elderly individuals from three cities in China and employed machine learning algorithms for predictive tasks, using univariate analysis methods for feature selection. Three machine learning algorithms were deployed for the predictive tasks, and the elderly medical and daily care services demand prediction models were developed and validated using 12 and 13 important features, respectively, including demographic, social, economic, health, and other parameters.

This investigation evaluated various models for forecasting healthcare services utilization among the elderly and identified LightGBM as the top-performing model, with an AUC of 0.910, F1-score of 0.841. Furthermore, LightGBM exhibited superior predictive capabilities (AUC = 0.906, F1-score = 0.819) when compared to other models in predicting the demand for elderly daily care services. Moreover, all predictive models exhibited high recall and precision, indicating that they can make accurate and efficient decisions regarding healthcare resource allocation and minimize costly risks. Related studies have demonstrated that LightGBM exhibit superior predictive performance in a range of applications^2,10,32^. For example, a study of environmental factors on the number of days demanded for cardiovascular admissions, which compared six machine learning models (including logistic regression (LR), support vector machine (SVM), ANN, random forest (RF), extreme gradient boosting (XGBoost), and light gradient boosting machine (LightGBM)), showed that LightGBM had the best performance among the integrated learning models^2^. Within the context of our research, the LightGBM model emerged as the superior predictor, with key variables including chronic diseases, education level, frequency of visit, and financial source emerging as the primary influencers of medical services utilization among the elderly. Concurrently, the model highlighted that the count of chronic diseases, education level, financial resources, monthly income, and frequency of visit were the key factors shaping the demand for daily care services within this demographic group. These findings align with our logistic regression analysis presented in the Supplementary Material, eTable 2. Educational level exerts a significant influence on the demand for medical and daily care services among the elderly, demonstrating a positive correlation. Specifically, elderly individuals with higher levels of education exhibit a stronger demand for medical and daily care services. This finding aligns with the results of Xu X^33^, suggesting that an elevated level of education is often associated with greater health awareness, attentiveness to personal health status, and a readiness to adopt new health-related concepts. As a result, these individuals tend to have higher expectations and requirements for healthcare services. The number of chronic diseases is a pivotal factor affecting the demand for medical and daily care services. Chronic conditions represent the most significant adverse influence on the quality of life for the elderly, particularly as age-related diseases are predominantly chronic, including cardiovascular and cerebrovascular diseases and diabetes mellitus. This is consistent with previous research identifying chronic diseases as a critical factor in the healthcare services demand among the elderly^34^. The presence of comorbidities or complications from chronic diseases can impair the elderly’s self-care capabilities^35^. Studies indicate that those aged 65 and over with chronic diseases often face reduced mobility and physical functioning, leading to an increased demand for medical and daily care services^36^. Additionally, our logistic regression results suggest that the frequency of visits is a determinant of the demand for medical services and daily care services among the elderly. Research indicates that the frequency of family visits may reflect the level of familial attention and support. Elderly individuals who receive fewer visits might feel insecure and neglected, prompting them to focus more on self-care and health maintenance^37^. Therefore, it is imperative for healthcare providers and policymakers to consider these factors when designing services aimed at improving the well-being of the elderly population.

Machine learning represents a significant advancement in the field of research, moving beyond the traditional focus on the observation of influencing factors. Instead, it emphasizes the construction of predictive models and the importance ranking of influencing factors. This approach offers valuable insights that can inform the formulation of relevant policies by the government in the context of an aging population. Furthermore, it can enhance the efficiency and cost-effectiveness of management, ultimately leading to more effective services that can alleviate the challenges posed by an aging population. Based on the findings of this study, the following recommendations are made: First, the service demands of vulnerable elderly people who are old, financially disadvantaged, and suffering from illness should be included in the basic elderly care services security system to ensure their basic living. On the one hand, the government has the responsibility to provide this basic security; on the other hand, given the limited payment capacity of vulnerable elderly groups, they cannot obtain necessary medical and daily care services on their own. Secondly, it is necessary to strengthen the construction of the basic medical services system, especially the support for vulnerable elderly groups, and further optimize the medical and daily care subsidy system, expand subsidy coverage, and increase subsidy funds by strengthening the assessment of their needs and capabilities. Finally, it is recommended to combine medical insurance with elderly care services to provide professional medical services for the elderly. In addition, barriers between elderly care and medical insurance should be eliminated to ensure that the elderly can obtain medical and daily care services without worries.

## Limitations

This study has some limitations. Firstly, this study is a cross-sectional study, which cannot dynamically reflect the change of data in the time series, and more data are needed subsequently to gain insight into the process and pattern of the demand for medical and daily care services for the elderly at home. Secondly, this study lacks medical clinical indicators of the elderly, which has some limitations in guiding the demand for medical and living services for the elderly. Finally, this study only used three algorithms to construct the prediction model, which does not entirely determine that a particular model is an optimal model, and more related research is still needed to select the most suitable prediction model for this field.

## Conclusion

In summary, this study, leveraging questionnaire data in conjunction with feature selection, techniques for addressing imbalanced data, and machine learning methods, has contributed to a deeper understanding of healthcare services utilization among the elderly. Furthermore, it has provided a predictive model that aids in confronting the challenges posed by population aging. The insights garnered from this research can inform the development of more effective and targeted healthcare policies and services, ultimately enhancing the well-being of the elderly population.

## Electronic supplementary material

Below is the link to the electronic supplementary material.

Supplementary Material 1

## Data Availability

The datasets used and/or analysed during the current study available from the corresponding author on reasonable request.
