# Supplementary material for: Utilizing SMOTE-TomekLink and machine learning to construct a predictive model for elderly medical and daily care services demand
Source: Sci Rep. 2025 Mar 11;15:8446. doi: 10.1038/s41598-025-92722-1 (PMC11897399; doi:10.1038/s41598-025-92722-1)
Supplement: Supplementary file 1 — Supplementary Material 1 [file 41598_2025_92722_MOESM1_ESM.docx]

eTable 1 Demand for Medical and Daily Care Services for the Elderly

| Variables | Number (n) | | Percentage (%) | |
| --- | --- | --- | --- | --- |
| Medical Services |  | |  | |
| 0 | 161 | | 12.5 | |
| 1 | 561 | | 43.5 | |
| 2 | 344 | | 26.6 | |
| ≥3 | 225 | | 17.4 | |
| Daily care service | |  | |  |
| 0 | | 377 | | 29.2 |
| 1 | | 322 | | 24.9 |
| 2 | | 133 | | 10.3 |
| ≥3 | | 459 | | 35.6 |

eTable 2 Logistic Regression Analysis of Demand for Medical and Daily Care Services for the Elderly

| Demand for medical services | |  | Demand for Daily care services | |
| --- | --- | --- | --- | --- |
| variables | *OR（95% CI）* |  | Variables | *OR（95% CI）* |
| Age (years) |  |  | Gender |  |
| 60~ | 1.000 |  | Male | 1.000 |
| 70~ | 1.469（0.876~2.462） |  | Female | 1.001（0.699~1.433） |
| 80~97 | 1.317（0.735~2.359） |  | Age (years) |  |
| Educational level |  |  | 60~ | 1.000 |
| Illiterate/Little Literacy | 1.000 |  | 70~ | 1.516（1.054~2.181） |
| Primary School | 2.789（1.596~4.872） |  | 80~97 | 5.576（3.365~9.239） |
| Middle School | 1.618（0.918~2.851） |  | Educational level |  |
| High school and above | 1.477（0.784~2.781） |  | Illiterate/Little Literacy | 1.000 |
| Financial Sources |  |  | Primary School | 1.136（0.740~1.745） |
| Pension | 1.000 |  | Middle School | 1.035（0.644~1.663） |
| Relative child allowance | 1.796（0.991~3.255） |  | High school and above | 1.220（0.724~2.054） |
| Labor income | 2.544（1.378~4.694） |  | Financial Sources |  |
| Other | 1.328（0.695~2.536） |  | Pension | 1.000 |
| Monthly income (￥) |  |  | Relative child allowance | 1.395（0.827~2.356） |
| ≤1000 | 1.000 |  | Labor income | 1.323（0.784~2.233） |
| 1001~3000 | 1.389（0.840~2.299） |  | Other | 1.114（0.629~1.973） |
| 3001~5000 | 3.827（1.920~7.627） |  | Monthly income (￥) |  |
| ≥5001 | 3.916（1.791~8.563） |  | ≤1000 | 1.000 |
| Medical Insurance |  |  | 1001~3000 | 0.745（0.497~1.116） |
| No | 1.000 |  | 3001~5000 | 1.730（1.041~2.875） |
| Basic medical insurance for urban and rural workers | 0.334（0.136~0.823） |  | ≥5001 | 1.306（0.757~2.251） |
| Basic Medical Insurance for Urban and Rural Residents | 1.212（0.544~2.698） |  | Medical Insurance |  |
| Others | 0.792（0.179~3.506） |  | No | 1.000 |
| Spouse |  |  | Basic medical insurance for urban and rural workers | 1.757（0.851~3.629） |
| No | 1.000 |  | Basic Medical Insurance for Urban and Rural Residents | 2.071（1.090~3.937） |
| Yes | 0.613（0.348~1.080） |  | Others | 2.582（0.904~7.377） |
| Number of children |  |  | Spouse |  |
| 0 | 1.000 |  | No | 1.000 |
| 1 | 0.328（0.055~1.951） |  | Yes | 0.400（0.250~0.642） |
| 2 | 0.386（0.065~2.284） |  | Number of children |  |
| ≥3 | 0.326（0.053~1.988） |  | 0 | 1.000 |
| Living Style |  |  | 1 | 0.808（0.312~2.089） |
| Lives alone | 1.000 |  | 2 | 0.785（0.305~2.022） |
| Living with family | 0.965（0.539~1.727） |  | ≥3 | 0.748（0.281~1.992） |
| Other | 2.959（0.880~9.944） |  | Living Style |  |
| Frequency of visits |  |  | Lives alone | 1.000 |
| Once a day | 1.000 |  | Living with family | 0.712（0.438~1.159） |
| Once a week | 1.518（0.970~2.378） |  | Other | 3.249（1.305~8.092） |
| Half a month or once a month | 2.882（1.515~5.482） |  | Frequency of visits |  |
| More than once every three months | 2.521（1.217~5.223） |  | Once a day | 1.000 |
| Smoking |  |  | Once a week | 1.378（0.950~1.999） |
| No | 1.000 |  | Half a month or once a month | 1.654（1.074~2.546） |
| Yes | 1.397（0.889~2.194） |  | More than once every three months | 0.999（0.593~1.684） |
| Sleeping time (h) |  |  | Smoking |  |
| <6 | 1.000 |  | No | 1.000 |
| 6~8 | 0.681（0.444~1.045） |  | Yes | 1.240（0.839~1.834） |
| >8 | 1.003（0.582~1.731） |  | Sleeping time (h) |  |
| Number of chronic diseases |  |  | <6 | 1.000 |
| 0 | 1.000 |  | 6~8 | 0.979（0.703~1.363） |
| 1 | 1.320（0.825~2.111） |  | >8 | 1.490（0.971~2.286） |
| 2 | 1.652（0.984~2.774） |  | Number of chronic diseases |  |
| ≥3 | 2.384（1.341~4.239） |  | 0 | 1.000 |
|  |  |  | 1 | 1.827（1.228~2.718） |
|  |  |  | 2 | 2.546（1.658~3.910） |
|  |  |  | ≥3 | 4.263（2.698~6.736） |
